# Redefining the Cervical Pedicle Entry Point (CPEP) in the Insertion of Cervical Pedicle Screw Using O-Arm Navigation: A Prospective Radiological Study From India

**DOI:** 10.7759/cureus.84372

**Published:** 2025-05-18

**Authors:** Bharat Dave, Sandesh Agarawal, Ajay Krishnan, Shivanand Mayi, Ravi Rai, Mirant B Dave

**Affiliations:** 1 Spine Surgery, Stavya Spine Hospital and Research Institute, Ahmedabad, IND; 2 Spine Surgery, Bhavnagar Institute of Medical Science (BIMS), Bhavnagar, IND

**Keywords:** accuracy, cervical pedicle entry point, cervical pedicle screw, o-arm-navigation, pedicle

## Abstract

Introduction

Cervical pedicle screw (CPS) fixation offers superior biomechanical stability in the management of cervical spine pathologies. However, the technique is associated with a significant risk of screw malposition due to the small pedicle dimensions and the lateralized trajectory of conventional entry points, often complicated by medial paraspinal muscular force. This study aimed to evaluate the safety and accuracy of a novel, medially-shifted cervical pedicle entry point (CPEP) for sub-axial CPS placement utilizing intraoperative O-arm™-based navigation (Medtronic, Minneapolis, USA).

Methods and materials

A prospective cohort study was conducted on 50 patients undergoing sub-axial CPS fixation between July 2021 and January 2023. All procedures employed an intraoperative O-arm imaging system integrated with the StealthStation S8 Navigation Platform (Medtronic, Minneapolis, USA). The CPEP was defined as the intersection of a vertical line bisecting the lateral mass and a horizontal line 2 mm inferior to its superior margin. Screw trajectory was planned and executed entirely under navigation guidance. Screw accuracy was assessed postoperatively using multiplanar computed tomography and classified according to the Neo grading system.

Results

A total of 218 CPSs were inserted using the proposed CPEP technique. The overall accuracy rate was 97.24%, with a total breach rate of 2.76%. Specifically, four screws exhibited Grade 1 breaches, one screw exhibited a Grade 2 breach, and one a Grade 3 breach; the latter two were revised intraoperatively. No intraoperative or postoperative neurovascular complications were observed.

Conclusion

The novel CPEP technique, characterized by a medially positioned entry point and executed under O-arm navigation, demonstrated high accuracy and safety in CPS placement. The approach effectively reduces soft tissue dissection and mitigates lateral malposition risks associated with conventional lateral entry points. These findings support the adoption of CPEP as a viable alternative to traditional CPS techniques, warranting further investigation in larger, multi-center cohorts.

## Introduction

Cervical pedicle screw (CPS) fixation has emerged as a biomechanically superior alternative to traditional lateral mass fixation, particularly in cases requiring robust multi-planar stability such as trauma, deformity correction, or revision surgery. Several cadaveric and in vivo biomechanical studies have consistently demonstrated that CPS constructs provide significantly greater pull-out strength, three-dimensional rigidity, and resistance to torsional, flexion-extension, and lateral bending forces when compared to lateral mass screws [1-4]. This enhanced biomechanical profile allows for more secure fixation, especially in osteoporotic bone or multilevel constructs, and facilitates early mobilization and better deformity correction [5-7]. Despite these advantages, widespread adoption of CPS remains limited due to technical difficulty and risks of neurovascular injury. Therefore, improving the safety and reproducibility of CPS placement remains a critical priority in cervical spine surgery [8]. However, ensuring a safe placement of CPS is significantly challenging, primarily due to the petite dimensions of the cervical pedicles [9].

Studies have demonstrated considerable convergence angle for cervical pedicle screws trajectory in the transverse plane that might pose obstacles during entry-canal preparation due to abundant posterior cervical muscles [10]. This increased convergence angle for CPS increases the risk of screw mal-positioning that increases the risk of violating the foramina transversarium laterally rather than the spinal canal medially. Leveraging advancements in technology, computer-assisted surgery has emerged as a promising tool for enhancing the accuracy of CPS insertion [11, 12]. In collaboration with evolving technology, particularly the integration of Intra-operative CT scans using the O-arm™ and navigation systems (Medtronic, Minneapolis, USA), we follow a protocol by introducing our cervical pedicle entry point (CPEP) for cervical pedicle screw insertion by shifting the entry point more medially than the conventional entry point. This innovative technique is proposed with the anticipation of mitigating muscle interference during screw canal preparation and thereby reducing the chances of catastrophic vascular complications especially when guided by navigation system. In addition, we evaluated accuracy of screw placement utilizing our novel CPEP technique.

We hypothesize that the proposed medially shifted CPEP, when utilized under O-arm-based intraoperative navigation, improves the accuracy of screw placement and reduces the risk of lateral malposition and soft tissue trauma. This approach may offer enhanced safety and ease of execution by minimizing the need for extensive muscle dissection and retraction. Simultaneously, using this technique under O-arm navigation guidance, on the other hand, reduces the risk of intraoperative vascular and neurological complications.

## Materials and methods

This single-center prospective study included 50 patients who underwent sub-axial CPS fixation under the guidance of intraoperative O-arm CT scan and navigation. Stavya Spine Hospital and Research Institute Institutional Ethics Committee issued approval (No. SSHRI/CS/II/CxPS/DD/36/03-21), adhering to the principles outlined in the Declaration of Helsinki. The study was conducted between January 2021 and January 2023. The study was registered with the Clinical Trials Registry - India, with CTRI number CTRI/2021/04/032866, dated 16/04/2021.

Patients aged over 18 years with degenerative cervical spinal stenosis associated with either instability or multilevel degenerative kyphosis were included in the study. These conditions were evaluated intraoperatively using a single helical cone-beam computed tomography (CbCT) system (O-arm™ O2 Imaging System, Medtronic, Minneapolis, MN, USA) in conjunction with a surgical navigation platform (StealthStation™ S8, Medtronic, Minneapolis, MN, USA). Patients with a history of congenital/developmental cervical spine malformations (i.e., hemivertebrae, block vertebrae, absence of pedicle), previous cervical spine surgery, or conditions such as neoplastic, infectious, or traumatic fracture of pedicle/vertebral body, potentially affecting pedicle morphology, were excluded from the study. During the insertion of cervical pedicle screws, the O-arm was utilized along with the StealthStation S8 navigation system. This integrated system provided both 2D fluoroscopic and full-3D CT reconstructions during surgical procedures for the data to smoothly transfer to the navigation system (StealthStation S8). In this study, all spinal images, including 3D reconstructions, were acquired to minimize potential errors associated with this process.

Surgical steps

**Figure 1:**
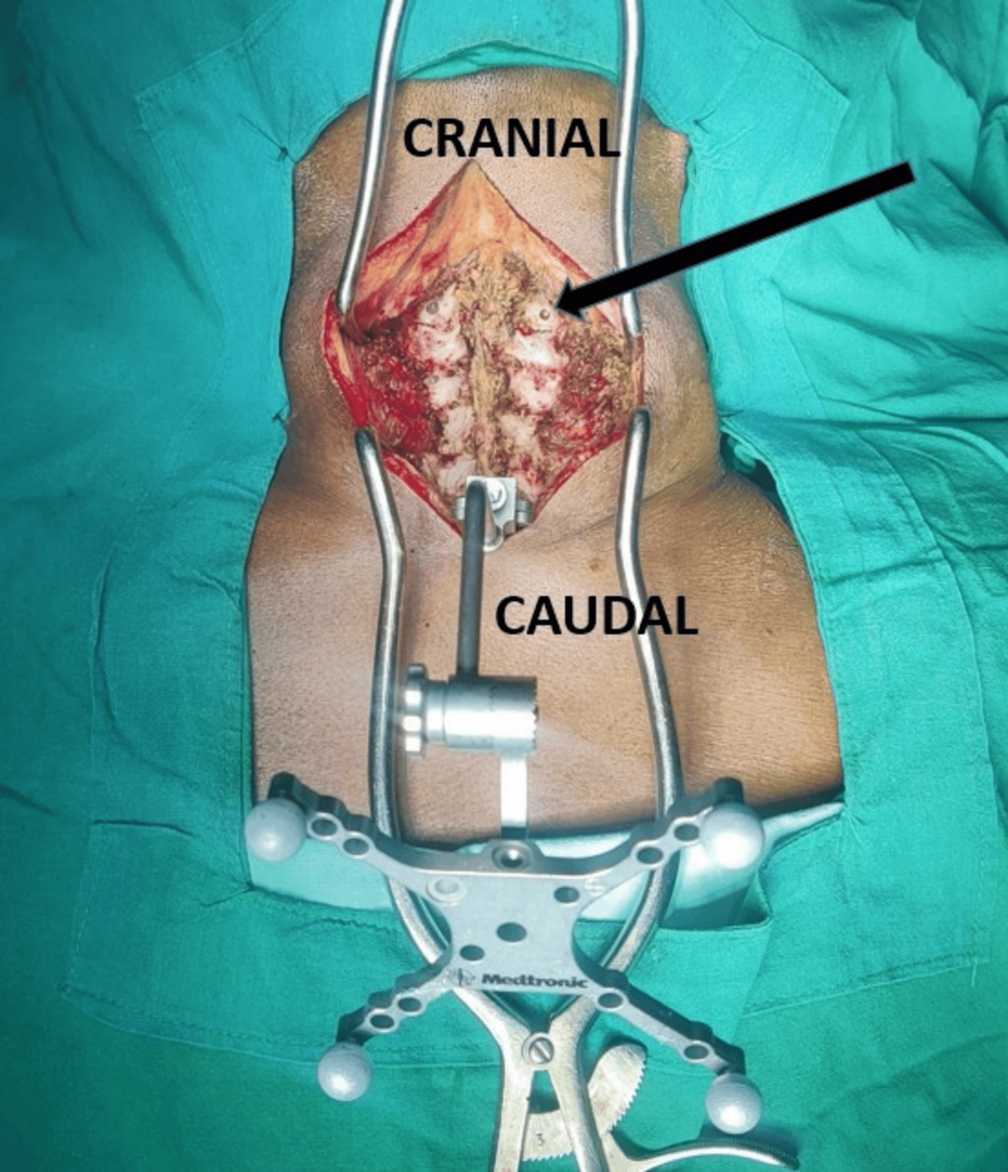
Clinical intraoperative image showing placement of Gelpi retractors and the navigation frame prior to taking an intraoperative CT scan. The black arrow points to the entry point for cervical pedicle screw prepared with a diamond burr tip.

All patients were operated in the prone position under general anesthesia with neck immobilization using Gardner-Well tongs traction. Using a posterior midline approach, bilateral exposure till the lateral border of the lateral mass was done to all the desired levels of surgery before CPEP insertion. After confirming proper alignment posteriorly, a navigation reference frame was affixed to the spinous process of C2 to get spanning images from C3 to C7, and 3D CT data were acquired using the O-arm. Once the navigation system was ready, the entry point for CPS insertion was determined according to our proposed entry point (CPEP). Instead of the traditional lateral entry point adjacent to the midpoint of the lateral mass, a slight medial entry point in the midline of the lateral mass was adopted for this study, which we call the cervical pedicle entry point (CPEP). Gelpi retractors were placed before acquiring the 3D CT scan and left undisturbed throughout the instrumentation procedure, as illustrated in Figure 1.

The probable projection of screws was planned and the retractors were placed in a way that they did not interfere with the screw placement. The navigation system facilitated trajectory planning and guidance for CPS insertion through CPEP. Initially, a funnel was created using a diamond burr tip of diameter 4 mm, followed by establishing an entry point with a match-head burr tip of diameter 2.2 mm. The trajectory was then tapped using a 2.5 mm tap, followed by insertion of 3.5- or 4-mm pedicle screw. It is important to note that all surgical steps were performed strictly under navigation guidance. Notably, the entry point guided by navigation was situated more medially than the conventional method, which helps mitigate lateral tissue dissection and retraction. Verification of pedicle wall integrity was performed using a navigation probe post trajectory establishment with a match-head burr tip. Prior to screw insertion, diameter and length of all cervical pedicles were assessed using a navigation probe. All the screws were initially inserted through half-way because full insertion could have changed the trajectory of the other screws. After confirmation with the second O-arm spin, the screws were further inserted and a rod-screw construct was created bilaterally. Any screw with a breach grade of >1 was promptly revised/removed.

The evaluation of the accuracy of CPS was calculated with a post-instrumentation CT in the multiplanar view based on the classification described by Neo et al. [13]. CPS positions were categorized into four grades based on the degree of pedicle wall breach: Grade 0 indicating no breach, with the screw completely contained in the pedicle; Grade 1 denoting breach < 2 mm; Grade 2 for breach 2-4 mm; and Grade 3 representing perforations ≥ 4 mm, indicative of complete breach.

## Results

Between July 2021 and January 2023, a cohort of 50 patients was included in the study. Among these 50-patient cohort, a total of 218 sub-axial cervical spine pedicles underwent CPS using CPEP, as shown in Table 1. Within this group, four CPS showed a Grade 1 breach, while one demonstrated a Grade 2 breach, and another showed a Grade 3 breach. Screws with Grade 2 and Grade 3 breach were redirected and reconfirmed with another CT spin. No revision of screw performed with Grade 1 breach and none of the screws was removed due to Grade 2 or 3 breach. Consequently, the accuracy of the navigation system in guiding cervical pedicle screw instrumentation was calculated at 97.24% (212 of 218), with an overall breach rate of 2.76% (six of 218).

**Table 1:** Distribution of screws and the grade of screw breach at individual cervical pedicle levels on post-instrumentation 3D-CT using O-arm. O-arm S8 Navigation System™ by Medtronic, Minneapolis, USA.

Pedicle	Number of screws	Number of breaches	Number of Grade 1 breaches	Number of Grade 2 breaches	Number of Grade 3 breaches
C3	26	2	1	1	0
C4	61	0	0	0	0
C5	60	3	3	0	0
C6	51	1	0	0	1
C7	20	0	0	0	0
Total	218	6	4	1	1

**Figure 2:**
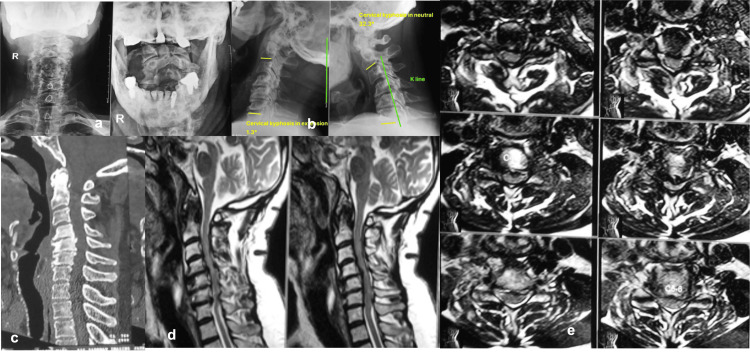
Preoperative pictures of a male patient with cervical myelopathy Preoperative pictures of a male patient with cervical myelopathy shows a) AP and Lateral X-ray, b) lateral flexion-extension X-rays, c) CT scan sagittal view showing disc-osteophyte complex with kyphosis, d) T2 sagittal view of MRI showing cervical stenosis and e) T2 axial view from C3-C7 levels showing narrowing of spinal canal diameter.

**Figure 3:**
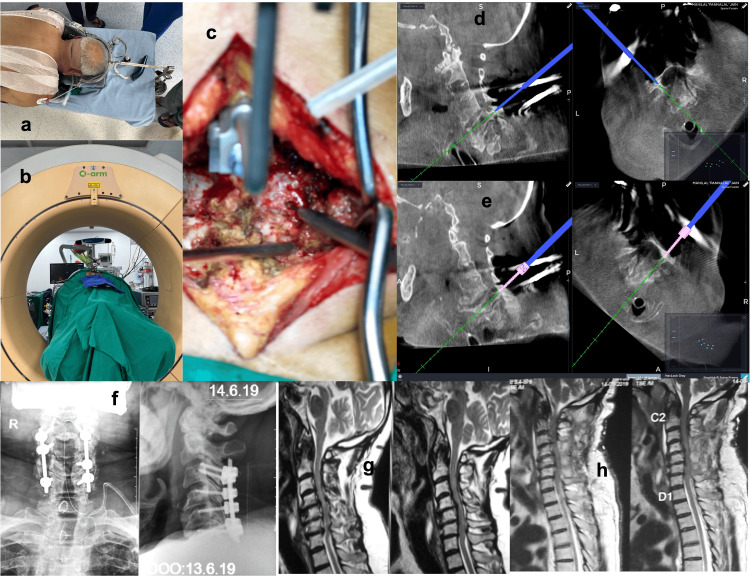
Intraoperative pictures of a male patient with cervical myelopathy Intraoperative pictures of a male patient with cervical myelopathy shows a) positioning and fixation of cervical spine with cervical tongs, b) Intraoperative CT cuts using O-arm and navigation, c) our CPEP entry point at cervical pedicle using matchhead burr, d) intraoperative cervical pedicle entry with cervical probe under navigation, e) insertion of pedicle screw with trajectory under navigation guidance, f) postoperative x-ray of cervical spine AP and Lateral view, g) preoperative T2 sagittal view of MRI comparing with h) postoperative T2 sagittal view of MRI showing improvement in kyphosis and spinal canal compression.

Importantly, none of the patients enrolled in the study experienced any major screw-related complications such as vascular (vertebral artery) injury or neurological deficit intraoperatively or postoperatively. There were no incidences of screw loosening or breakage of the rod during the final follow-up. Figure 2a-2e depicts preoperative X-rays, CT scan, and MRI images of one of the representative cases of a 67-year-old male patient with cervical myelopathy with kyphosis. Figure 3a-3h depicts the same case with intraoperative positioning, O-arm navigation CT cuts, planning of CPEP entry point with burr and navigational images for pedicle screw insertion and the final X-ray and MRI images with good correction in kyphotic deformity.

## Discussion

The unique and complex anatomy of the cervical spine, such as unique cervical vertebral body structure, small and complex cervical pedicles, and the close proximity of the pedicles to critical neural and vascular structures, may be challenging for successful pedicle screw insertion. Therefore, number of researchers have proposed various methods for cervical pedicle screw placement [14, 15, 16]. The technological support of 3-dimensional computer-assisted navigation is a big leap forward with an improved accuracy of 89%-100% in thoraco-lumbar pedicle screw fixation.

**Figure 4:**
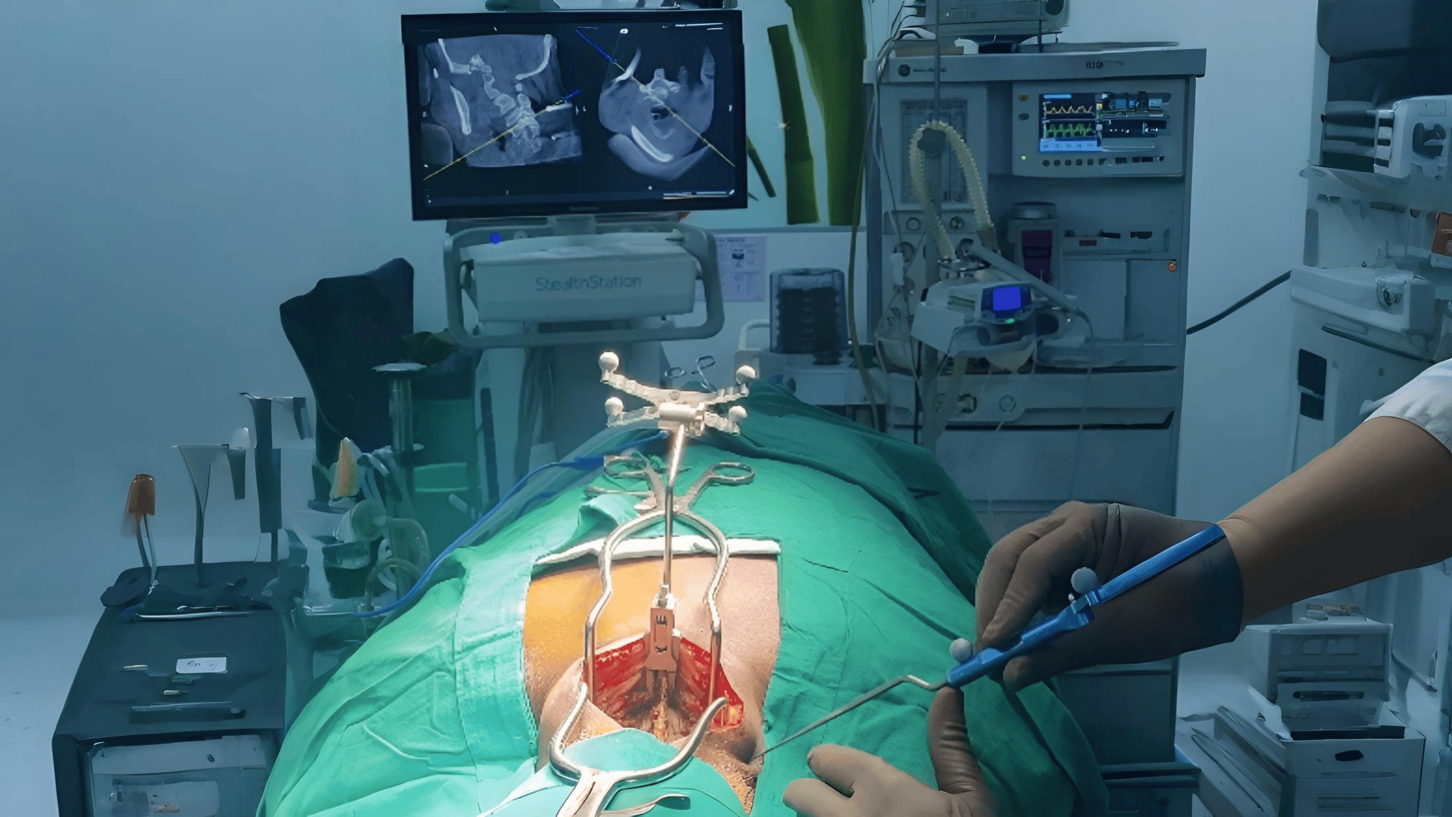
The trajectory of the instruments and the probable amount of tissue retraction required when inserting the screw via the traditional method.

**Figure 5:**
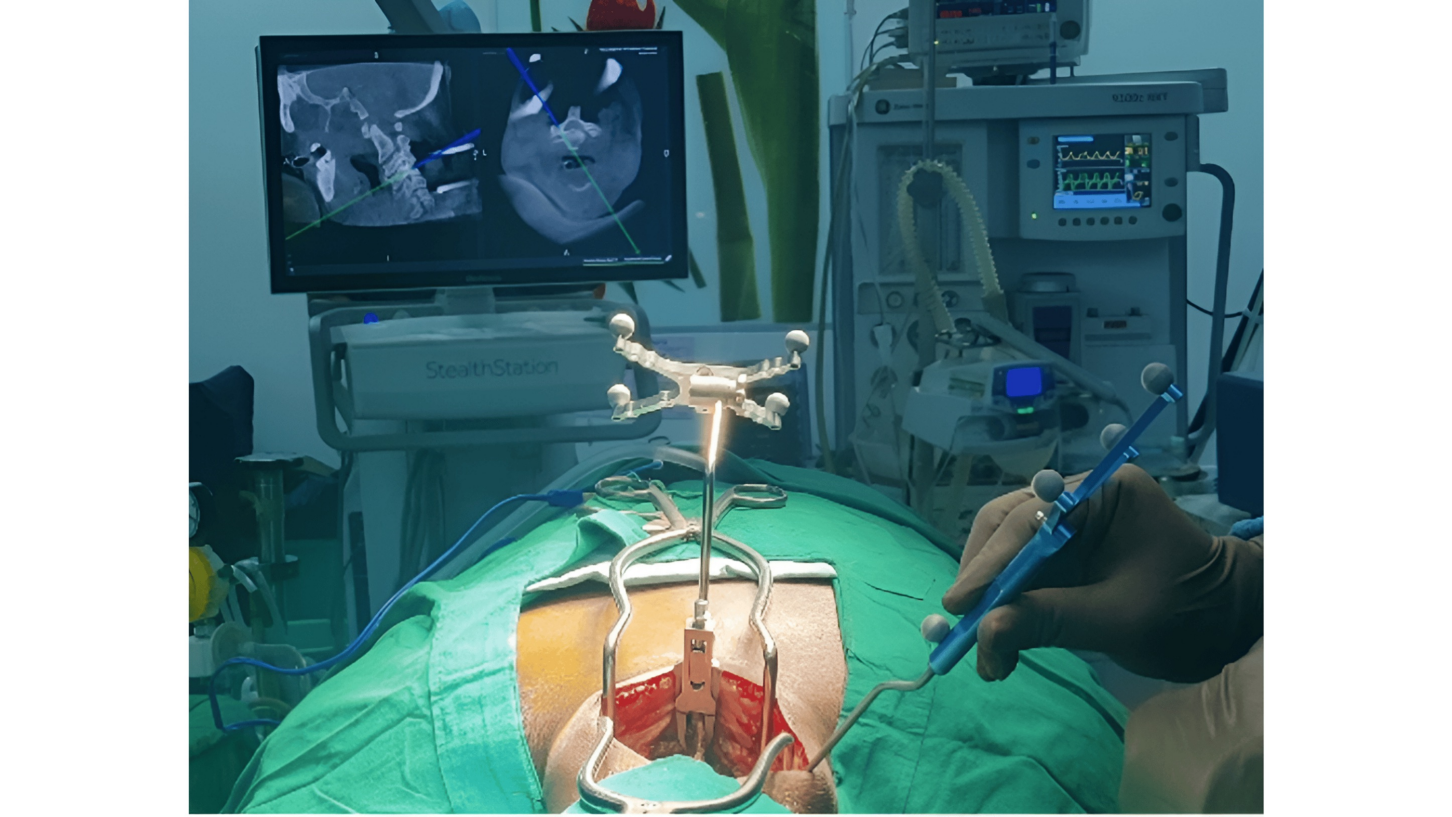
The trajectory of instruments and tissue retraction required with the entry point proposed by our study.

Ishikawa et al. reported two cases of cervical pedicle screw placement where the navigation system was used, and they found the incidence of penetrating the cortical bone was 11.1% and 18.7%, respectively. They noted that the penetration was more severe than the traditional manual screw fixation with radiographical monitoring [17, 18]. Possible reasons for decreased accuracy rate for the computer assisted navigation CPS could be because 1) the neck is in suspension during posterior surgery (unlike the temporarily fixed state of the thoracolumbar vertebrae), 2) the orientation of cervical pedicle angulation (pedicle transverse angle), and 3) the need of excessive lateral dissection and muscular retraction laterally required during the procedure. The deeply situated cervical spine and bulk of musculature may interfere with the trajectory of instruments, especially when utilizing the conventional entry point for CPS, which is situated more lateral to the mid-line of the lateral mass, as described in Figure 4 and Figure 5, respectively.

**Figure 6:**
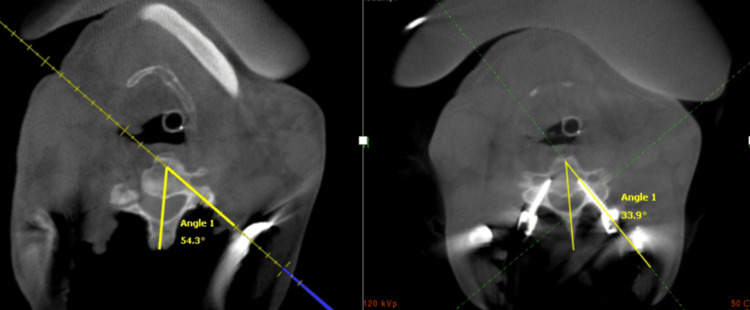
A comparison between the angle of trajectory by a navigation probe while utilizing the conventional entry point (left) and the angle of trajectory of a screw placed through the entry point proposed by our study (right).

The stronger the axial trajectory is kept against the medial pressure over the paracervical muscles, the easier the axial rotation or sagittal bending of the cervical spine, which can increase the chances of lateral mal-positioning of CPS. To overcome the obstacles of the CPS technique due to paracervical muscles, we used Gardner-Well tongs to hold the cervical spine stable. Additionally, two Gelpi retractors were placed at the cranial and caudal ends of the surgical field, which were left undisturbed until the completion of CPS insertion. This caution helped reduce navigation errors that can occur due to soft tissue retraction because the navigation system is a virtual imaging modality and not real-time fluoroscopy. Using the technology advantage of intraoperative CT scans with the O-arm and navigation system, we proposed a protocol of shifting the entry point (EP) more medially (CPEP) than the traditional CPS protocol, which improved the accuracy of CPS to 97.24% in our cases, as described in Figure 6. All screws were inserted through the same midline incision as against separate stab incisions.

Various methods and different entry points have been proposed for CPS insertion in the literature. Abumi et al. chose the entry point to be slightly lateral to the center of the articular mass and close to the posterior margin of the superior articular surface [14]. Lee et al. proposed the entry point 2 mm directly medial to the most lateral notch [19]. All these proposed entry points lie lateral to the midline/midpoint of the lateral mass, and hence, they require significant muscle retraction laterally. When they are used with the navigation system, they can lead to errors due to interference with the instrument trajectory. The entry point proposed by our study lies along an imaginary vertical line bisecting the lateral mass into two equal medial and lateral halves and a horizontal line 2 mm below the superior margin of the lateral mass in the coronal plane. In the sagittal plane, it was angled cranially in the case of C3 and C4, perpendicular in the case of C5, and caudally in the case of C6 and C7 CPS.

The CPEP proposed in our study helped us reduce the amount of soft tissue dissection and retraction laterally, which improved the accuracy rate of CPS because the medial force coming to the surgical instruments due to paracervical muscles was mitigated. We agree that the length of the screw might be few millimeters shorter in our CPEP than the conventional CPS technique due to high convergence angle. However, according to the literature, the strength of pedicle screw depends upon the purchase of the screw in the pedicle, and hence, a shorter screw was acceptable with our CPEP entry point [20]. In fact, we believe that our CPEP technique would reduce lateral breach of the pedicle screws due to the force directly coming medially due to strong paracervical musculature, which is the most positive aspect of our technique. 

While this study primarily focuses on the accuracy of pedicle screw placement, we also hypothesize that the CPEP technique, by reducing lateral muscle retraction, may lead to a decrease in surgical trauma, potentially improving postoperative recovery and minimizing pain. This reduction in muscle retraction could result in a more favorable postoperative recovery trajectory, enhancing functional outcomes and reducing postoperative discomfort. However, we acknowledge that this is a hypothesis and that further clinical studies with long-term follow-up data are required to validate these potential clinical benefits. This warrants future research to evaluate the clinical outcomes, such as pain scores, functional recovery, and quality of life, in conjunction with the technical advantages of the CPEP technique.

Limitations

This is a single-center, single-surgeon study with a relatively small sample size of Indian subjects. Therefore, the findings may not be representative of a broader and diverse patient demographic, which limits the acceptability of the results across the other groups. We could not include a comparative analysis with traditional methods of CPS insertion, and therefore, it is not possible to prove the superiority of the proposed CPEP method over the existing techniques. In spite of advancements in spine surgery technology, we must remember that challenges may arise during the integration and utilization of intraoperative CT scans with O-arm and navigation systems. Technical issues or system malfunctions could potentially impact the accuracy and reliability of the surgical procedure. However, since our results show an accuracy of 97.24% in using CPS with CPEP, we feel that our technique is unique and can provide further guidelines for future research. In addition, we feel that our technique can also be used under C-arm guidance.

## Conclusions

The present study introduces a novel cervical pedicle entry point (CPEP) technique for cervical pedicle screw (CPS) insertion, which addresses the anatomical and technical challenges associated with traditional methods. The integration of intraoperative O-arm-based navigation significantly enhanced the accuracy of CPS placement while minimizing the need for extensive soft tissue dissection, offering a potential advantage in reducing surgical trauma. The surgical methodology, combined with detailed radiological assessment, ensures reproducibility and offers the potential for adoption by other spine centers. This modified entry-point technique demonstrated a high accuracy rate of 97.24% with a low cortical breach rate of 2.76%, indicating its feasibility, safety, and reproducibility.
